# The BDNF Val66Met Polymorphism Has No Effect on Encoding-Related Hippocampal Response But Influences Recall in Remitted Patients With Bipolar Disorder

**DOI:** 10.3389/fpsyt.2019.00845

**Published:** 2019-12-06

**Authors:** Lone Diana Hørlyck, Julian Macoveanu, Maj Vinberg, Lars Vedel Kessing, Hartwig Roman Siebner, Kamilla Woznica Miskowiak

**Affiliations:** ^1^Neurocognition and Emotion in Affective Disorders (NEAD) Group, Copenhagen Affective Disorder Research Centre (CADIC), Copenhagen Psychiatric Centre, Copenhagen University Hospital, Rigshospitalet, Copenhagen, Denmark; ^2^Department of Psychology, University of Copenhagen, Copenhagen, Denmark; ^3^CADIC, Copenhagen Psychiatric Centre, Copenhagen University Hospital, Rigshospitalet, Copenhagen, Denmark; ^4^Danish Research Centre for Magnetic Resonance (DRCMR), Centre for Functional and Diagnostic Imaging and Research, Hvidovre Hospital, University of Copenhagen, Copenhagen, Denmark; ^5^Department of Neurology, Copenhagen University Hospital Bispebjerg, Copenhagen, Denmark

**Keywords:** hippocampus, cognitive impairment, affective disorders, fMRI, BDNF val66met genotype

## Abstract

**Background:** Cognitive impairments in bipolar disorder (BD) such as memory deficits are associated with poor functional outcomes and it has been suggested that the brain-derived neurotrophic factor (BDNF) Val66Met polymorphism contributes to individual variability in memory function in BD. The current study investigated the relationship between the BDNF Val66Met polymorphism, neural activity during a picture-encoding task, and subsequent memory recall.

**Methods:** A total of 70 patients with BD grouped according to genotype [ValVal or Met carriers (MetVal/MetMet)] underwent fMRI while performing a picture-encoding task. Memory for the encoded pictures was tested with a subsequent free recall memory task.

**Results:** There was no difference between the ValVal homozygotes and Met carriers in the involvement of hypothesized memory encoding regions i.e. hippocampus and dorsal prefrontal cortex (dPFC). However, an exploratory whole-brain analysis showed greater encoding-related lateral occipital cortex activity in Met carriers. Behaviorally, Met carriers also showed better free recall of the encoded pictures.

**Conclusions:** We found no effect of the BDNF genotype on encoding-related hippocampal and dPFC activity in BD, although Met carriers showed superior memory performance after the scan, which could be related to more efficient perceptual processing during encoding.

## Background

Cognitive impairments in bipolar disorder (BD) are associated with reduced functional capacity and poor prognosis (1). Specifically, patients’ verbal memory and executive function are among the strongest predictors for vocational capacity (2). The pattern of cognitive impairment in BD is heterogeneous, as some patients remain cognitively intact while up to 60% present with selective or global impairments (1). It is likely that genetic factors play a role in this heterogeneity, but the contribution of risk genotypes to the cognitive impairments in BD is poorly understood. It is important to gain a better understanding of these relationships and the factors that contribute to the cognitive heterogeneity seen in BD in order to offer personalized treatments and identify new therapeutic targets.

Several candidate vulnerability marker genes believed to confer risk of cognitive deficits in psychiatric disorders have been explored using cognitive assessments and imaging genetics (3). These include the Val66Met single nucleotide polymorphism in the gene encoding brain-derived neurotrophic factor (BDNF) and its relationship with memory function. In the Val66Met polymorphism, a valine to methionine substitution at codon 66 results in a switch from guanine to adenine at position 196 in the pro-region of the BDNF gene, leading to reduced activity-dependent BDNF secretion and potential associated changes in hippocampal functions such as episodic memory (4, 5).

There is some evidence suggesting that the Val66Met polymorphism affects cognition and neural processing, although findings from existing studies are mixed (6–10). A systematic review of studies using clinical and healthy populations found that 23 of 63 studies showed a significant relationship between memory and Val66Met carrier status (11). For example, Egan et al. (10) found reduced free recall memory for an auditory short story in Met carriers, which is known to rely on the hippocampus, although this effect of genotype was not present on a second measure of free recall [California Verbal Learning Test (CVLT)]. Some studies have also reported relationships between Val66Met and aspects of memory that are known to be less dependent of the hippocampus such as recognition memory (12, 13). However, in a study using a similar task to the task used in the current study, Dodds et al. (6) presented healthy participants with scene pictures during encoding and asked participants to make indoor/outdoor judgments of the pictures as they were presented. Subsequently, participants completed a recognition memory test indicating whether the presented pictures were old or new. This study found no differences in memory performance or neural activity during encoding between BDNF Val66Met genotypes among these healthy individuals. However, subsequent studies using similar paradigms have found both reduced (9) and increased (8) MTL activity during memory encoding, and hence, inconsistencies in studies with healthy participants remain.

Given the albeit mixed evidence for impact of BDNF genotype on memory performance and related neural processing across healthy and psychiatric populations, it is pertinent to consider that BDNF genotype might play a role in the cognitive heterogeneity seen in BD but only few studies have investigated the relationship between memory and Val66Met genotype in BD. One study showed that Met carriers with BD displayed reduced verbal memory performance (and smaller hippocampal volumes) compared to both healthy and depressed Met carriers, suggesting a specific disadvantage for Met carrier BD patients on memory function (14). Another study using a more general screening for cognitive function including memory did not find an association with Val66Met status (15). To our knowledge, no studies have investigated the neural underpinnings of the association between BDNF Val66Met genotype and memory performance in BD, which may provide a more sensitive measure of the relationship between genotype, memory function, and neural mechanisms.

In this study, we investigated the effects of BDNF genotype on encoding-related hippocampal and prefrontal cortex (PFC) activity and memory retrieval in a strategic picture-encoding task in 70 patients with BD in full or partial remission. We used a picture encoding task identical to Dodds et al. (6) but used a free recall memory task to assess memory performance instead of a recognition task. Free recall of complex scenes relies heavily on the hippocampus (16) and also the PFC (17, 18) and these neural mechanisms appear to be specific for free recall over for instance item memory (19–21). Further, studies have indicated a possible advantage of Met carriers in terms of PFC function and working memory (22–24). For instance, one study showed that over-expression of BDNF in mice resulted in decreased working memory function (23). Also, altered PFC activity has been associated with cognitive deficits in BD (25, 26). Hence, we hypothesized that i) Met carriers would show reduced hippocampal and/or increased dorsal PFC (dPFC) activity during picture encoding and ii) this altered activity would be associated with impaired free recall for the encoded pictures.

## Materials and Methods

### Participants

Seventy BD patients in partial or full remission were included in the study. Baseline data for the patients was obtained from patients’ participation in two randomized intervention studies investigating cognitive function in BD (27, 28). Participants were between 18 and 65 years of age (mean ± SD; 37 ± 10) and had normal or corrected-to-normal vision. All participants were screened with Schedules for Clinical Assessment in Neuropsychiatry (SCAN) to confirm diagnosis of BD. Participants were also rated on the Hamilton Depression Rating Scale [HDRS-17; (29)] and Young Mania Scale [YMRS; (30)] to confirm that they were in partial or full remission (HDRS and YMRS scores ≤14). The original studies were approved by the local ethics committee, Danish Data Agency, and Danish Medicines Agency, and written consent was obtained from all participants prior to beginning of the study. For further details on the recruitment and screening processes, please see previous studies (27, 28).

### Genotyping

Genomic DNA was extracted from blood samples with the Maxwell Blood DNA purification kit (Promega, Madison, WI, USA) in accordance with the manufacturer’s protocol and the samples were genotyped using the Illumina Infinium PsychArrayBeadChip (Illumina, San Diego, CA, USA).

### Magnetic Resonance Imaging

fMRI data was collected at the Danish Research Centre for Magnetic Resonance with an eight-channel head coil on a 3T Siemens Trio MR scanner (Siemens, Erlangen, Germany). Blood oxygen level-dependent T2*-weighted functional images were acquired using echo-planar imaging (EPI) with the following parameters: repetition time (TR), 2490 ms; echo time, 30 ms; slice thickness, 3 mm; field of view (FOV), 192 × 192 mm using a 64 × 64 grid, flip angle 20°. A total of 117 volumes were acquired in a single fMRI session and each volume consisted of 42 slices. A field-map was recorded to allow distortion correction of the acquired EPI images. Participants also underwent a high-resolution structural scan where a T1-weighted 3D structural image was acquired to co-register with the functional images, with the following parameters: TI = 800, TE = 3.93, TR = 1540 ms, flip angle 9°, 1 × 1 × 1 mm voxel size, 256×256 FOV, and 192 slices.

### fMRI Data Analysis

#### Pre-Processing

Pre-processing was carried out with the fMRI Expert Analysis Tool (FEAT; version 6.0) in FMRIB’s software library (FSL; www.fmrib.ox.ac.uk/fsl). Standard pre-processing steps included non-brain removal, realignment, correction for B0 field distortions, slice time correction, and spatial normalization of functional and structural images to a Montreal Neurologic Institute (MNI) template. The normalized functional images were spatially smoothed using an isotropic Gaussian smoothing kernel with a full width at half maximum (FWHM) of 5 mm. The time series in each session was high pass filtered (to max. 0.008 Hz).

#### Statistical Analysis

At the first level, picture encoding and rest events were modelled as boxcar functions and convolved with a canonical hemodynamic response function. A picture encoding BOLD contrast was computed by subtracting non-encoding periods from the picture encoding events.

**Figure 1:**
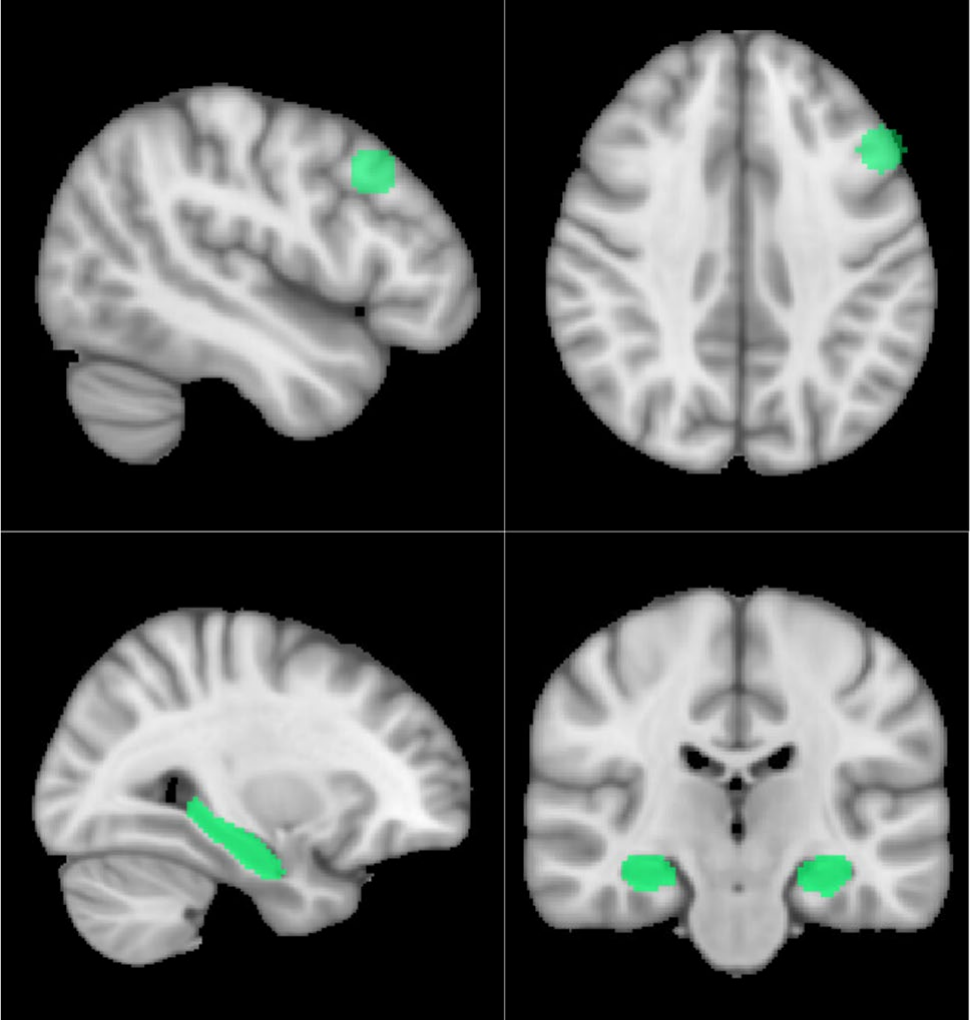
Visualization of the dPFC (above) and hippocampal (below) ROIs.

At group level, we assessed differences in the picture-encoding contrast task between the two genotype groups. The group-level statistical estimation was carried out using nonparametric permutation tests with the FSL Randomise tool (http://www.fmrib.ox.ac.uk/fsl/randomise/index.html, 31) using default settings and 5000 permutations. This method was used as it has been shown that permutation testing produces nominal results for clusterwise inference, while parametric statistical methods failed to do so (32). ValVal and Met carrier genotype groups were contrasted for each of the pre-determined regions of interest (ROIs). The ROIs were constructed on the MNI template and included bilateral hippocampi and the dPFC. The hippocampal ROI was anatomically defined, while the dPFC ROI was a spherical ROI based on a previous paper investigating dPFC activity during encoding and subsequent free recall (33) (**Figure 1**). The bilateral hippocampus was defined using cortical maps thresholded at 30% provided by the Harvard-Oxford cortical structure atlas implemented in FSLView (34). In addition to these ROI analyses, we conducted an exploratory whole-brain analysis applying a brain mask to investigate whether genotype groups’ encoding-related activity differed in any other (unforeseen) regions. For analyses at the group level, demographic variables including age, sex, years of education, and clinical variables (HDRS and YMRS) were modelled as regressors of no interest. The resulting data were assessed using Threshold-Free Cluster Enhancement (TFCE) to identify potential clusters (35).

Finally, we extracted the encoding-related BOLD signal change from the predefined ROIs and any additional regions showing differential response between genotype groups and correlated these values with participants subsequent recall score.

### Picture Encoding and Retrieval Task

Stimuli pictures consisted of images of complex scenes collected from the International Affective Picture System [IAPS; (36)]. Several studies have demonstrated that encoding of such complex images is related to hippocampal activity (9, 37). All images were matched for valence, arousal, and complexity. Participants received instructions on the screen prior to the beginning of the task. During fMRI, participants viewed six 24-s picture blocks, each block consisting of six pictures. Each picture was presented on the screen for 3 s followed by an inter-stimulus interval (ISI) of 1 s in a pseudo-randomized order. Each picture block was followed by a short rest in which participants saw a fixation cross presented in the middle of the screen for 24 s. The total duration of the task was approximately 5 min. Participants were instructed to pay careful attention to the presented pictures, as they would be required to complete a subsequent memory task. Hence, the task comprised a strategic memory-encoding component. Participants were also instructed to make indoor-outdoor judgments for each picture, ensuring that participants paid attention to the task. Each picture block contained an equal number of indoor and outdoor scenes.

Immediately following the scanning session, participants completed a free recall test of the pictures, in which participants were instructed to tell the experimenter about as many pictures they could remember and describe each picture in detail. One point was awarded for each remembered picture, yielding a total free recall score.

### Statistical Analysis

Analyses of behavioral data and extracted mean percent signal change from the identified clusters in the FSL Randomise analysis were performed using SPSS (version 22; IBM Corporation).

As only a small number (N = 4) of participants carried the MetMet genotype, ValMet and MetMet carriers were pooled together in a Met-carrier (MetCar) group (N = 26) and compared to ValVal homozygotes (N = 43). Performance on the picture memory task was calculated as the number of correctly recalled images on the free recall test. For each participant, a raw score was calculated with a score of 1 being given for each remembered picture. The total number of false positives was also recorded. ValVal vs MetCar genotypes were compared for demographic and clinical variables and performance on memory tests using independent samples t-tests (two-tailed) and χ^2^. The relationship between neural activity in ROIs/significant clusters and memory performance was assessed with Pearson’s correlations and Fisher’s r to z transformation. Likewise, the relationship between clinical measures, age, years of education, memory recall, and neural activity was assessed with Pearson’s correlations. Interaction effects between genotype and total number of medications on memory recall and encoding-related activity was assessed with a univariate ANOVA.

For all tests, it was ensured that assumptions were met, and where assumptions were violated, data were either square-root transformed to fit a Gaussian distribution or analyzed with non-parametric tests (Mann-Whitney U). The alpha-level was set at P = 0.05.

## Results

Of the 70 participants recruited for the study, two participants had missing behavioral data and were omitted from fMRI analyses, yielding N = 68 subjects (ValVal: N = 42; ValMet/MetMet: N = 26) for analysis. There were no differences between the ValVal and the ValMet/MetMet genotype groups in demographic or clinical variables including age, years in education, age at onset, illness duration, number of hospitalisations, mood symptoms, or medication status (P > 0.05; **Table 1**).

**Table 1:** Means (SD) for demographic and clinical variables, picture memory task performance, and significance levels for differences between the ValVal and the ValMet/MetMet groups in remitted patients with bipolar disorder.

Variable	Genotype		P-value
	ValVal (N = 43)	ValMet/MetMet (N = 27)	
Age, mean (SD)	36.2 (10.3)	37.6 (10.7)	0.58
Years of education, mean (SD)	15.5 (3.2)	15.0 (3.4)	0.51
Age at onset	20.0 (9.3)	21.15 (9.6)	0.67
BD type, no type 1 (%)	18 (48.6)	13 (61.9)	0.33
Illness duration, mean (SD)	16.8 (8.9)	16.8 (11.4)	0.98
No of depressive episodes, mean (SD)	6.1 (5.9)	7.3 (8.7)	0.55
No of manic episodes mean (SD)	8.7 (8.5)	7.4 (10.8)	0.61
No of hospitalisations, mean (SD)	2.7(0.8)	2.7 (0.8)	0.78
Gender, no women (%)	60.0	56.5	0.99
HDRS-17 baseline, mean (SD)	7.6 (5.1)	7.9 (4.8)	0.75
YMRS baseline, mean (SD)	2.1 (2.4)	2.5 (2.4)	0.33
Picture Memory score, mean (SD)	8.0 (5.3)	10.3 (5.0)	0.05
			
Medication			
Lithium n (%)	17 (45.9)	11 (55.0)	
Anticonvulsants, n (%)	23 (62.2)	11 (55.0)	0.60
Antidepressants, n (%)	14 (37.8)	8 (40.0)	0.87
Antipsychotics, n (%)	14 (37.8)	6 (30.0)	0.51
Benzodiazepines, n (%)	10 (27.0)	5 (25.0)	0.87
Melatonin, n (%)	1 (2.7)	2 (10.0)	0.24
No of medications, means (SD)	2.16 (0.8)	2.10 (1.0)	0.80

HDRS-17, Hamilton Depression Rating Scale 17 items; YMRS, Young Mania Rating Scale; SD, standard deviation.

### Functional MRI Analysis

#### Main Effect of Task

**Figure 2:**
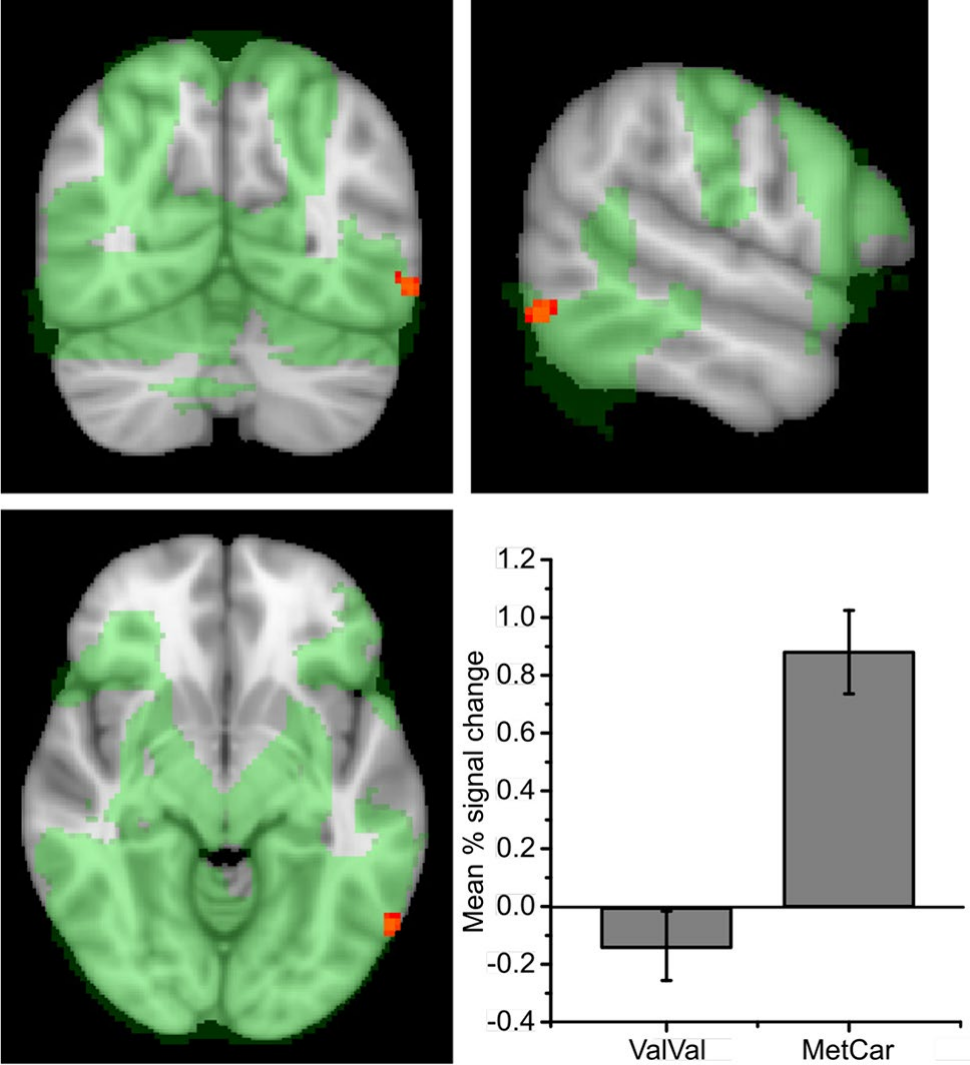
Encoding activity during encoding across all participating remitted patients with bipolar disorder, showing activity in a large network (marked in green). Cluster shows encoding activity for ValMet/MetMet > ValVal (-56 -64 -10). Effects displayed on an MNI template and thresholded at P > 0.05 (TFCE whole-brain). Error bars show SE. Plot shows mean percent blood-oxygen dependent (BOLD) signal change during encoding within the significant cluster. Error bars show standard error (SE).

To confirm that the task was associated with the expected activity in visual areas and the hypothesized ROIs, we carried out a non-parametric one-sample t-test across all subjects showing that picture encoding was associated with activity in a large network of brain regions, including the dorsal PFC, occipital, parietal, and temporal regions (see **Figure 2**; network shown in green). Across all participants, picture encoding vs. baseline was also associated with increased activity in the predefined ROIs: the bilateral hippocampi and the dPFC (TFCE corrected P < 0.05).

#### Effect of Genotype

In contrast to our hypothesis, there were no significant effects of genotype on encoding-related neural activity in the bilateral hippocampus, or in the dPFC at P = 0.05 (TFCE) in the ROI analyses. An exploratory whole-brain analysis showed that Met carriers displayed greater activity in a cluster located to the lateral occipital (LO) cortex compared to participants with the ValVal genotype (peak coordinate: x = –58 y = –66, z = –8; **Table 2**; **Figure 2**).

**Table 2:** Significant clusters for the picture encoding task. Cluster peak and local maximum is represented with x, y, z MNI coordinates and Threshold-Free Cluster Enhancement corrected P-values.

Task and Region	Side	Cluster TFCE P	Cluster size (voxels)	x	y	z
Picture encoding						
Occipital cortex	L	P < 0.002	192	–58	–66	–8

L, left; MNI, Montreal Neurologic Institute; R, right; TFCE, Threshold-Free Cluster Enhancement.

#### Relationship Between Neural Activity and Memory Performance

Across all subjects, there was no statistically significant correlation between hippocampal activity (mean % signal change) and memory recall, r(68) = 0.07, P = 0.56. There was also no statistically significant difference in this correlation between the ValVal (r = -0.07, P = 0.65) and the Met carrier (r = 0.05, P = 0.80) groups, Z = - 0.47, P = 0.64. In the dPFC, there was no statistically significant correlation between memory recall and mean % signal change, r(68) = 0.10, P = 0.42, although when comparing the correlations for the two genotypes, these were significantly different, Z = 2.33, P = 0.02, which was due to a positive correlation between mean % signal change in the dPFC and memory for ValVal [r(42) = 0.29, P = 0.06], while this correlation was negative for Met carriers, r(24) = -0.31, P = 0.13. We also investigated the relationship between memory recall and the activity in the identified significant cluster in the occipital lobe across all subjects but this correlation was also non-significant r_s_(68) = 0.17, P = 0.17. Furthermore, there was no difference in correlation between activity in the significant cluster and memory recall between the two groups, Z = 0.65, P = 0.51.

### Behavioral Analyses

**Figure 3:**
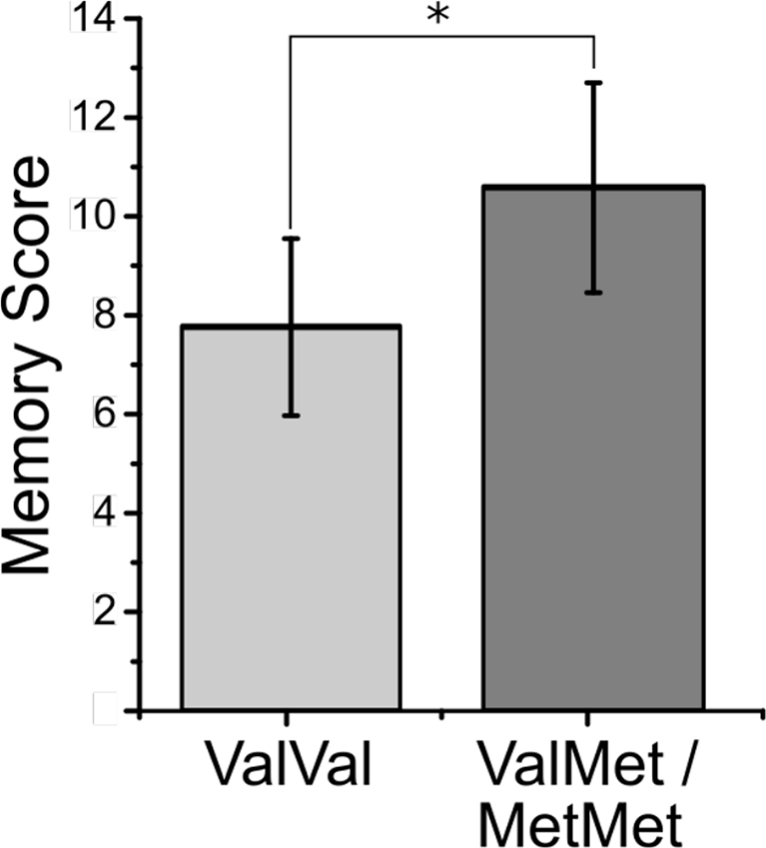
Mean memory score on the picture memory task for the ValVal and the ValMet/MetMet groups in remitted patients with bipolar disorder. Error bars represent SE. Significance of asterisk is at the 0.05 level (exact p-value is P = 0.02).

Picture recall after the scan: An independent samples t-test carried out on the square root-transformed data showed that unexpectedly, Met carriers displayed a superior picture memory performance (higher number of correctly recalled pictures) in comparison with Val homozygotes [mean ± SD, Met carriers: 10.3 ± 5.0, Val homozygotes: 8.0 ± 5.3; t(67) = 2.35, P = 0.02, d = 0.61; **Figure 3**]. There were no differences between the two groups in the number of false positives (i.e., “made up” pictures) (U = 437.0, P = 0.71, η^2^ = 0.002). There were no significant correlations between picture recall and sub-syndromal mania or depression symptoms, age, and years of education in either of the two groups or across the entire sample (P ≥ 0.20). Use of antidepressants, lithium, anticonvulsants, antipsychotics, and diazepines was not associated with memory recall (P ≥ 0.13). There was no significant interaction effect between val66met BDNF genotype and total number of medications on memory recall (P = 0.23). The main effect of medication was also non-significant (P = 0.27).

### Associations Between Mood, Medication and BOLD Response

To investigate whether associations with mood or medication status could explain our obtained fMRI results, we carried out the following analyses: Across the entire sample, subsyndromal depression symptoms showed a negative correlation with mean % signal change in the bilateral hippocampus *(HDRS*; *r* = -0.25, *P* = 0.04). This effect was due to a strong relationship between HDRS scores and hippocampal activity within the ValVal group (r = -0.49, P < 0.001), while there was no significant relationship between HDRS scores and hippocampal activity in the Met carrier group alone (r = 0.28, P = 0.17). For the dPFC and the identified cluster in the LO cortex, there were no significant relationships between mean % signal change in these regions during encoding and HDRS or YMRS scores across the entire sample (P’s > 0.20) or within the genotype groups (P’s > 0.19).

Use of antidepressants, lithium, anticonvulsants, antipsychotics, and diazepines was not associated with mean percent BOLD signal change in the hippocampal or dPFC ROIs or in the identified LO cortex cluster (P’s ≥ 0.05). Assessing the effects of the total number of medications on neural activity during encoding, there was no significant interaction between val66met BDNF genotype and total number of medications for encoding-related activity in the dPFC or hippocampal ROIs, or in the identified LO cortex cluster (P > 0.05).

### Sensitivity Analyses

To rule out the effects of subsyndromal symptoms in the current analyses, we conducted a sensitivity analysis only including the participants in full remission (HDRS < 7; YMRS < 7), yielding a total of 34 datasets for analysis.

Like in the full dataset, participants that were carriers of the Met allele showed numerically better memory performance (M = 10.17, SD = 4.51) compared to ValVal homozygotes (M = 7.62, SD = 4.40), although this effect did not reach significance for this subset of participants, t(31) = 1.59, P = 0.12. Consistent with the analyses conducted on all participants, for fMRI analyses, there was no significant effect of genotype on activity during encoding in the bilateral hippocampal ROI or the dPFC. An exploratory whole-brain analysis did also not show any significant effect of genotype on activity during encoding.

## Discussion

We investigated for the first time the effects of BDNF Val66Met genotype on encoding-related hippocampal response and memory performance in partially or fully remitted patients with BD. In contrast to our hypothesis, we found no impact of this BDNF genotype on hippocampal (or dorsal PFC response) during picture encoding. However, an exploratory whole-brain analysis showed that Met carriers displayed greater encoding-related neural activity in the LO cortex compared to ValVal homozygotes. At a behavioral level, Met carriers also displayed superior picture recall following the encoding session in the scanner compared to the ValVal homozygotes.

The lack of an association between BDNF genotype and hippocampal activity during encoding is in accordance with a previous study on healthy volunteers using an identical picture encoding task (6) but contrasts with other similar studies showing either no difference (9) or increased (8) MTL activity during encoding of complex scenes in healthy subjects. The lack of a healthy control group in this study to serve as a baseline challenges the interpretation of the current findings in relation to existing studies in healthy volunteers and hence comprises a limitation of the current study. Nevertheless, investigating the relationship between BDNF genotype, memory, and neural mechanisms in BD alone can still be used to identify potential mechanisms underlying the heterogeneity in cognitive impairment across BD patients. In this context, our results do not suggest that differences in hippocampal processing during memory encoding between Met carriers and ValVal homozygotes contribute to cognitive heterogeneity in BD.

In the exploratory whole-brain analysis, we observed an effect of BDNF Val66Met genotype on neural processing in a cluster located to the LO cortex, with Met carriers showing greater activity during encoding in this region compared to ValVal homozygotes, indicating a potential difference in visual processing that is dependent on genotype. The lateral occipital cortex is known to be involved in visual processing and object perception and recognition (38) and has specifically been implicated in identifying object shapes (39). Why encoding-related processing in this area should be greater in Met carriers than ValVal homozygotes is not clear, but this finding is consistent with the observed better memory recall in the Met carrier group, although we did not observe a significant relationship between the mean percent BOLD signal change extracted from the identified cluster and subsequent memory performance.

Surprisingly, we observed better picture recall in BD Met carriers than in ValVal homozygotes. This was in contrast to our hypothesis that Met carriers would show reduced memory performance and also contradicts the majority of studies reporting a relationship between BDNF genotype and memory performance, where higher Met load is often associated with poorer memory in both healthy volunteers (9, 11, 12) and BD (14, 40). In terms of comparison with results from studies using healthy volunteers, we expected that potential effects of Val66Met BDNF genotype would be exacerbated in BD. However, it is possible that residual BD symptoms might instead blunt potential differences due to BDNF genotype. Another possibility is that different methods of memory assessment explain the divergent findings; we used a free recall task of complex scenes whereas others investigating the relationship between BDNF genotype and memory in BD have used other tasks such as the CVLT (41, 42). Hence, it is possible that subtle differences in the mechanisms supporting different memory types might have played a role in the divergent findings, where Met carriers may be impaired compared to ValVal homozygotes on a verbal learning task but not a visual learning task, which is consistent with our finding showing increased activity during encoding in the LO cortex known to play a role in visual processing.

Also, some studies have suggested that there might be a possible advantage of Met carriers in terms of PFC function and working memory (22). Free recall is dependent on both PFC processing and working memory function and we did see a significant positive correlation between dPFC activity during encoding and subsequent memory for the Met carrier group that was not present in the ValVal group. Hence, it is possible that Met carriers more efficiently recruited the dPFC during encoding.

Another point is that the previous studies showing better performance for ValVal homozygotes compared to Met carriers in psychiatric populations have often used samples including both remitted and currently depressed patients (e.g. 15). Such samples may show reduced performance on cognitive tasks due to patients’ affective symptoms (43), which might interact with Met load. A strength of the current study was the inclusion of a relatively homogeneous sample of remitted or partially remitted BD patients, which limited confounding effects of affective symptoms compared to previous studies. However, this study did include patients in both partial and full remission, and hence, sub-syndromal mood symptoms could have had an impact on memory performance in this study too. In this context, we found a negative correlation between residual depression symptoms and hippocampal activity during encoding across the entire sample, suggesting that patients with more depressive symptoms recruited the hippocampus less efficiently during learning. This observation is consistent with the state-related impairments in learning and memory seen in depression. Interestingly, this association was more pronounced in the ValVal homozygotes who are believed to have more activity-dependent hippocampal BDNF trafficking and greater encoding-related hippocampal response when healthy (9).

To further address the potential significance of residual depressive symptoms, we carried out sensitivity analyses only including patients in full remission and consistent with the main analyses, we found no significant effect of genotype on activity during encoding in the predefined dPFC or hippocampal ROIs. For the sensitivity analyses, the whole-brain analyses did not yield any significant effect of genotype. However it should be noted that the sample size was dramatically reduced in these analyses (N = 33).

For the main analyses, we used a relatively large sample size (N = 68) for assessment of differences between the genotype groups in encoding-related neural activity. In comparison, other studies used between 22 and 58 participants (6, 8, 9, 14, 42). Due to a small number of participants with the MetMet genotype (N = 4), we decided to not compare patients with the MetMet and ValMet genotypes. We were thus unable to explore a potential association between Met load and clinical, cognitive and neural responses as done in previous studies (6, 44). Indeed, this could have brought more clarity to the current results showing a weak advantage for Met-carriers on the free recall task. Further, as all participants received psychotropic medication it cannot be excluded that this has influenced the present result. Finally, as mentioned previously, having a healthy control group to serve as a baseline and comparison with previous studies using healthy subjects would have been an advantage.

In conclusion, we found no association between BDNF val66met genotype and hippocampal or dorsal PFC activity during picture encoding, although Met carriers showed an unexpected greater picture recall after the scan. Exploratory whole-brain analysis revealed larger lateral occipital cortex response in Met carriers, which might reflect increased visual processing during picture encoding in this group, although activity in this region did not correlate with subsequent memory performance. Instead, Met carriers might show more efficient processing in the dPFC during encoding compared to ValVal carriers. The absence of effects of BDNF val66met genotype on memory-related hippocampal response in our BD sample may be due to the effects of subsyndromal symptoms overriding more subtle effects of BDNF val66met genotype since we allowed subsyndromal symptoms in our sample in the interest of generalizability. Specifically, future studies should use even larger samples (n > 70), include a healthy control group, and investigate fully remitted BD patients (with scores <7 on HDRS and YMRS) or healthy, non-medicated participants at high risk for BD (first-degree relatives) to assess potential dose-dependent effects of Met load on neural and cognitive measures of learning and memory in BD.

## Data Availability Statement

The raw data supporting the conclusions of this manuscript will be made available by the authors, without undue reservation, to any qualified researcher.

## Ethics Statement

The studies involving human participants were reviewed and approved by De Videnskabsetiske Komitéer, Region Hovedstaden. The patients/participants provided their written informed consent to participate in this study.

## Author Contributions

KM designed the original studies together with LK, MV, and HS. KM was responsible for carrying out the data collection. LH and JM analyzed the data under the supervision of KM. LH and KM wrote the manuscript. All authors approved the final version of the manuscript for submission.

## Funding

The study was based on two original trials funded by the TrygFonden, Danish Council for Independent Research, Novo Nordisk Foundation, Beckett Fonden, and Savværksejer Juhl’s Mindefond. The sponsors had no role in the planning or conduct of the study or in the interpretation of the results.

## Conflict of Interest

KM declares having received honoraria from Lundbeck and Allergan in the past three years. MV has received consultancy fee from Lundbeck A/S within the past three years. LK has within the preceding three years been a consultant for Lundbeck and Sunovion.

The remaining authors declare that the research was conducted in the absence of any commercial or financial relationships that could be construed as a potential conflict of interest.
